# Effect of a double Y-shaped miniplate over traditional miniplate in treating mandibular anterior fractures

**DOI:** 10.6026/973206300220620

**Published:** 2026-02-28

**Authors:** Soumyadev Satpathy, Nilabh Kalita, Shahidkhan Khalilahmed Pathan, Manav Ahuja, Gurleen Kaur, Sanjeev Mittal, Vardharajula Venkat Ramaiah

**Affiliations:** 1 Department of Dentistry, Fakir Medical College, Balasore, Odisha, India; 2 Intern, Government Dental College, Dibrugarh, Assam, India; 3 General Dentist, Thorncliffe Overlea Dental Center, Canada; 4 Department of Oral and Maxillofacial Surgery, ITS Dental College and Hospital, Moradnagar, India; 5 Intern, Kalinga Institute of Dental Sciences, KIIT Deemed to be University, Patia, Bhubaneswar-751024, Odisha, India; 6 Department of Prosthodontics, Maharishi Markandeswar College of Dental Sciences and Research, Mullana, Ambala, Haryana, India; 7 Department of Public Health, College of Medical Sciences, Qassim University, Buraydah 51542, PO, Box 666, Saudi Arabia

**Keywords:** Anterior fracture, conventional, double Y shaped manipulate, fixation, mandible

## Abstract

Open reduction and internal fixation are typically the preferred treatments for mandibular syphilis and parasymphysis fractures. Therefore, it is of interest to compare the effectiveness of conventional manipulates with double Y-shaped manipulates in treating mandibular anterior fractures. Hence, a total of twenty-two patients were split into two groups at random, each consisting of eleven samples: Group I underwent fracture fixation using traditional two manipulates, whereas Group II underwent fracture fixation using a double Y-shaped manipulate. Thus, we show that anterior mandibular fractures can be successfully treated with both traditional miniplates and double Y-shaped miniplates. Excellent fracture stability was shown by both plates.

## Background:

The largest and strongest bone in the face is the mandible. One of the most frequent skeletal injuries to the face is a mandibular fracture. Road traffic accidents, assaults, industrial injuries, falls and sports injuries can all cause them, though the proportion of each varies greatly among nations and regions. One of the most frequent locations for fractures is the mandibular body, which is followed by the condyle, angle, symphysis, ramus and coronoid process [1]. Due to its prominent location on the face, it is frequently fractured following maxillofacial trauma [2]. Despite the fact that nearly four times as much force is needed to fracture the mandible as the maxilla, it has been shown that mandibular fractures occur twice as frequently as mid-facial fractures [3]. Due to its fracture, the patient is unable to preserve aesthetics and engage in daily tasks such as speech, mastication and deglutition [2]. A considerable percentage of facial traumas are caused by mandibular fractures, with the mental foramen, the third molar angle area and the mandibular canine sites being especially prone to fracture due to the bone density in these areas. About 20% of mandibular fractures are caused by fractures through the mandible at the level of the symphysis and parasymphysis [4, 5]. Restoring appropriate alignment, stability and jaw function while accelerating healing and avoiding complications is the primary objective of treatment mandibular fractures [6]. The placement of bone fragments in their anatomical positions determines how a mandibular fracture is treated. One established risk factor for infection and non-union is movement at the fracture site. Segment reduction, stabilisation and immobilisation were the traditional methods used to treat fractures [4]. In order to treat mandibular fractures, locking plate/screw plating systems were introduced. These systems work on the principle of restricted back out and serve as internal fixators. They achieve stability by locking the screw to the plate, which has the special benefit of eliminating the need for the plate to come into close contact with the underlying bone, making plate adaptation simpler [2]. Treatment for mandibular fractures has significantly improved thanks to developments in medical science and technology. Traditionally, IMF was used in conjunction with arch bars, elastics and different wire techniques [6]. Early attempts at plate and screw fixation had several failures, most likely due to a lack of understanding of the biomechanics of these systems. These plates have numerous drawbacks, including as the possibility of damage to the teeth and neurovascular bundle from the bicortical screws. The patients found them to be heavy and uncomfortable [7]. Miniplates are currently the recommended surgical technique for the fixing of fractures and osteotomies. They have been utilised to promote stability between bone fragments in the maxillofacial region. Using miniplates has the benefit of being simple to handle, shape and adjust to the bone. Miniplate osteosynthesis facilitates early functional mobilisation, enhances bone healing and guarantees sufficient fracture stability [1]. Using Champy's concepts, transoral implantation of noncompressive miniplates-often referred to as "miniplates"-has recently gained popularity [8]. Due to their ease of handling and adaptability, miniplates have been used extensively for decades to treat mandibular fractures. For front mandibular fractures, lag screws have been reported as a dependable, stable and secure internal fixation technique [9]. Using Champy's concepts, transoral implantation of noncompressive miniplates-often referred to as "miniplates" has recently gained favour. The primary drawback of traditional bone plate/screw systems is that, in order to avoid changes in segment alignment, the plate must to be precisely matched to the underlying bone [10]. For elective healing, fracture parts in an anatomic bony union must undergo open reduction and internal fixation (ORIF). Existing laceration, extra-oral and intra-oral methods are utilised to visualise and reduce mandibular parasymphysis and symphysis fractures [4]. Double Y-shaped miniplates offer more resistance to displacement than traditional straight miniplates, according to a biomechanical study [11]. In order to address the shortcomings associated with the use of single four/six-hole miniplates and double miniplates, advanced osteosynthesis designs were introduced. As a result, a variety of plate shapes, including delta, lambda, trapezoidal, V, X, double Y and more, were created. These plates were made to take up less space while still distributing stress throughout the zones of compression and tension. These developing osteosynthesis materials offer three types of force resistance: torsion, shearing and bending [12]. Mandibular fractures have also been fixed using the 3D plating method. 3D plates are a time-saving substitute for traditional miniplates since they simultaneously stabilise the tension and compression zones [13]. Therefore, it is of interest to compare the effectiveness of conventional miniplates with double Y-shaped miniplates in treating mandibular anterior fractures.

## Materials and Methods:

After receiving institutional ethical committee permission and participant informed agreement, this study was conducted at the oral and maxillofacial surgery department. The study included patients who were admitted to the Department of Oral and Maxillofacial Surgery with a recent anterior mandibular fracture that required open reduction and stiff internal fixation. The inclusion and exclusion criteria were followed when conducting the study. For the study, 22 individuals with anterior mandibular fractures between the ages of 20 and 50 who were willing to participate were chosen. Patients with ASA classifications III, IV, V and VI, fractures older than two weeks, active infections at the fracture site, comminuted fractures and any pathology at the fracture site, diabetes and heart diseases were all excluded from the study. A total of 22 patients were split into two groups at random, each containing 11 samples. Group II received a double Y-shaped titanium miniplate with 30020 x 3 holes, fastened with monocortical screws, while Group I used a conventional two miniplate technique, plating fractures with a 2 mm, four-hole miniplate at the bottom border. The planned surgical procedure and any possible consequences were communicated to each patient. A preoperative clinical and radiographic assessment was conducted for two types of fractures: displacement and occlusion. Along with the subjects' demographic profile, preoperative occlusion, jaw mobility and discomfort status were noted. Maximum interincisal mouth opening and fracture segment stability are part of the post-operative examination. Using hands' thumb and index fingers, the bimanual palpation approach was used to assess stability. The study made use of the armamentarium and standardised materials. Patients were readied for surgery following standard blood tests, radiographic evaluations and prophylactic antibiotic coverage. Depending on convenience, either general anaesthesia or local anaesthesia was used for all procedures. If necessary, an Erich arch bar was placed on the mandibular and maxillary teeth. Following sufficient local anaesthesia, the fracture site was surgically exposed. In group I, the fracture was fixed using traditional miniplates, which included a 2 mm four- hole miniplate in the mandibular lower border and a 2 mm two-hole miniplate subapically, which were fastened with monocortical screws. Within Group II, A 5 mm gingival cuff was left in place after the fracture site was exposed and reduced using an intraoral vestibular incision. Six monocortical screws were used to secure the 2mm double Y-shaped miniplate used for fracture fixation. The occlusion was reevaluated after fixing. After haemostasis was reached, betadine solution was used to irrigate the surgical site. Layers of 3-0 Vicryl were used to close the wound. At three and six months, clinical and radiographic evaluations were performed on all patients. Post- operative mouth opening, plate adaptation and, handling, any complications and stability of fracture segment was done at baseline, 3month and 6 months of follow up. The obtained data was statistically evaluated using SPSS statistical software version 23.0 of IBM using independent t test and Chi square test at p<0.05.

## Results:

Table 1 indicates no changes in plate adaptation and handling among both groups. Table 2 indicates that mouth opening was improved significantly in groups II compared to Group I after 6 months of follow up. There was no significant difference among both groups post surgically with respect to post-operative complication, occlusion and stability (Table 3, Table 4, Table 5). There was only one complication seen in Double y shaped miniplate method of plating over 2 cases in conventional method. Table 6 indicates significant improvement in pain score from baseline to 6 months and on inter group comparison it was significant in group II compared to Group I.

## Discussion:

The contemporary method of open reduction and internal fixation of these fractures has been impacted by our growing understanding of the biomechanics and fracture healing of the mandible [5]. Using OPG obtained three and six months after surgery, our study demonstrated the good anatomic reduction of both conventional miniplates and double Y-shaped miniplates. The adoption of a twin Y plate fixation technique for anterior mandibular fractures is the main topic of this study. This innovative take on the traditional Y plate seeks to improve stability and biomechanical support for the fractured parts while reducing issues and postoperative complications [6]. Melek found that Y-shaped miniplates produced superior results when compared to lag screws in the treatment of anterior mandibular fractures [9]. Srivastava and Balakrishnan assessed the efficacy of double Y-shaped titanium plates and conventional miniplates in treating mandibular anterior fractures. They came to the conclusion that treating anterior mandibular fractures with a double Y-shaped titanium plate is a promising alternative to traditional methods [6]. The outcomes align with our conclusions. In the repair of anterior mandibular fractures, it has been demonstrated that Double Y-shaped miniplates have the same stabilisation because of their configuration as traditional mini plates [13]. Agrawal evaluated the application of Champy's miniplates and 2.0-mm stainless steel locking miniplates for mandibular fractures. They came to the conclusion that using a 2.0mm locking plate system contributes to greater stability [2]. In a study comparing 2.0mm regular miniplates with locking miniplates, Saikrishna *et al.* found that the locking plate/screw system was more rigid than the traditional plate/screw system [14]. Oral germs frequently cause mandibular fractures. The patient's innate reluctance to swallow or move his tongue freely increases the risk of infection, leading to stasis and the subsequent build-up of material in the fractured area. According to Zachariades *et al.* stability is thought to be the strongest defence against infection because movement in the presence of foreign objects, such as loose screws, typically results in infection and pseudoarthrosis. Additionally, it has been demonstrated that the intraoral method reduces infection rates [15]. The drawback of the present study was smaller sample size. Further studies are necessary to verify these findings with larges sample size. Awareness about oral hygiene, traumatic injuries and prevention of traumatic injuries should be crated among public [16, 17, 18-19]. For mandibular fractures, double Y-shaped miniplates are superior to conventional ones because they are simpler and quicker to adjust, use fewer screws, take less time during surgery, may improve stability and load distribution and cause less soft tissue irritation and nerve manipulation. Gouthami *et al.* concluded that for clinical application, the double "Y" miniplate and traditional 4-hole miniplates are equally effective [20]. Bhatt *et al.* came to the conclusion that, in comparison to traditional miniplating systems, 3D miniplates are a novel type of plating system with improved intraoperative and postoperative outcomes and more stability [21].

## Conclusion:

We show that anterior mandibular fractures could be successfully treated with both the traditional miniplate and the double Y-shaped miniplate. Six to eight monocortical screws are needed for fracture fixation using traditional two miniplates; however six monocortical screws are sufficient to hold a double Y-shaped plate. Comparatively speaking, the double Y-shaped miniplate approach was superior to the traditional one.

## Figures and Tables

**Table 1:** Plate handling and adapting type

**Ease of plate adaptation and handling**	**Conventional 2 miniplates (GROUP I)**	**Double y shaped miniplate (GROUP II)**	**p**
	**No. of Patients/ Percentage**	**No. of Patients/ Percentage**	
Good	12 (100%)	10 (83.3%)	0.364
Satisfactory	0	2 (16.7%)	
Total	12(100%)	12	
Test used-Chi square test,
significance-insignificant

**Table 2:** Mouth Opening (In Mm)

**Mouth opening (in mm)**	**Conventional 2 miniplates (GROUP I)**	**Double y shaped miniplate (GROUP II)**	**p**
	**Mean±SD**	**Mean±SD**	
Immediate Post OP	15.22± 0.876	14.78± 1.543	0.001
3 Month POD	33.14± 1.028	37.53± 1.134	
6 Month POD	39.14±0 .757	41.54± 0 .875	

**Table 3:** Post-operative complications

**Complications**	**Conventional 2 miniplates (GROUP I)**	**Double y shaped miniplate (GROUP II)**	**p**
	**Mean±SD**	**Mean±SD**	
Absent	10 (83.3%)	11(91.7%)	0.324
Present	2 (16.7%)	1 (0.8.3%)	
Total	12(100%)	12 (100%)	

**Table 4:** Post-operative occlusion

**Occlusion**	**Conventional 2 miniplates (GROUP I)**	**Double y shaped miniplate (GROUP II)**	**p**
	**Mean±SD**	**Mean±SD**	
Satisfactory	12 (100%)	10 (83.3%)	0.315
Mildly deranged	0	2 (16.7%)	
Total	12(100%)	12(100%)	

**Table 5:** Post- operative stability

**Occlusion**	**Conventional 2 miniplates (GROUP I)**	**Double y shaped miniplate (GROUP II)**	**p**
	**Mean±SD**	**Mean±SD**	
Stable	12 (100%)	10 (83.3%)	0.315
Unstable	0	2 (16.7%)	
Total	12(100%)	12(100%)	

**Table 6:** Post-operative pain score in both groups

**Pain score**	**Conventional 2 miniplates (GROUP I)**	**Double y shaped miniplate (GROUP II)**	**p**
	**Mean±SD**	**Mean±SD**	
Pain after 24 hours	2.48±0.36	2.01±0.57	0.05
Pain after 1 week	0.91±0.64	0.29±0.34	0.05
Pain after 1 month	0.12±0.42	0.03±0.27	0.05
